# Universal versus conditional three-day follow up visit for children with uncomplicated fever at the community level: design of a cluster-randomized, community-based, non-inferiority trial in Tanganyika, Democratic Republic of Congo

**DOI:** 10.1186/s12887-017-0792-1

**Published:** 2017-01-26

**Authors:** Elburg van Boetzelaer, Lara S. Ho, Julie R. Gutman, Laura C. Steinhardt, Alison Wittcoff, Yolanda Barbera, Pascal Ngoy, Steven A. Harvey, Luke C. Mullany

**Affiliations:** 1 International Rescue Committee, Kalemie, Democratic Republic of Congo; 2 International Rescue Committee, New York, USA; 3 Malaria Branch, Division of Parasitic Diseases and Malaria, Centers for Disease Control and Prevention, Atlanta, GA USA; 4 Department of International Health, Johns Hopkins Bloomberg School of Public Health, 615 N. Wolfe Street, W5009C, Baltimore, MD 21205 USA

**Keywords:** Non-inferiority, Pediatrics, Uncomplicated fever, Integrated community case management, Community health worker, Cluster randomized trial, Democratic Republic of the Congo

## Abstract

**Background:**

The current recommendation within integrated Community Case Management guidelines that all children presenting with uncomplicated fever and no danger signs be followed up after three days may not be necessary. Such fevers often resolve rapidly (usually within 48–96 h), and previous studies suggest that expectant home care for uncomplicated fever can be safely recommended. We aim to determine the non-inferiority of a conditional versus a universal follow-up visit for these children.

**Methods:**

We are conducting a cluster-randomized, community-based, non-inferiority trial enrolling ~4300 children (ages 2–59 months) presenting to community health workers (CHWs) with uncomplicated fever in Tanganyika Province, Democratic Republic of the Congo. Clusters (*n* = 28) of CHWs are randomized to advise caretakers of such children to either 1) return for a follow-up visit on Day 3 following the initial consultation (Day 1), regardless of illness resolution (as per current guidelines) or 2) return for a follow-up visit on Day 3 only if the child’s signs have not resolved. Enrolled children are followed up at Day 7 for a repeat assessment and recording of the primary outcome of the study, “failure”, which is defined as having fever, diarrhea, pneumonia or decline of health status (e.g. hospitalization, presenting danger signs, or death).

**Discussion:**

The results of this trial will be interpreted in conjunction with a similarly designed trial currently ongoing in Ethiopia. If a follow-up visit conditional on continued illness is shown to be non-inferior to current guidelines stipulating universal follow-up, appropriate updating of such guidelines could reduce time and human resource pressures on both providers and caregivers throughout communities of sub-Saharan Africa and South Asia.

**Trial registration:**

This trial was registered at ClinicalTrials.gov (NCT02595827) on November 2nd, 2015

## Background

In settings burdened with elevated under-five morbidity and mortality, and challenged by limited access to health care services, community health workers (CHWs) often provide first-level care for sick children aged 2–59 months directly in the community through the implementation of integrated community case management (iCCM) of common childhood illnesses. Current World Health Organization (WHO) iCCM guidelines prescribe that a febrile child presenting to a CHW be assessed for malaria, pneumonia, and diarrhea, and for general danger signs (e.g. child is unable to drink or breast feed, is vomiting everything, is having convulsions, is lethargic or unconscious, and/or has chest in-drawing). CHWs provide direct treatment for confirmed malaria, diarrhea, and pneumonia, and, if any danger sign is present, refer the child immediately to a health center for more extensive care. However, when a febrile child has none of the aforementioned illnesses or danger signs, the CHW provides the child with an antipyretic and *advises the caregiver to return to the CHW for a follow-up consultation on Day 3* [1].

Such uncomplicated childhood febrile illnesses are common, are often due to viruses, and in many cases resolve rapidly and spontaneously (i.e. often within 48 h, and almost always within 96 h) [2–5]. In a study of 986 mRDT-negative children at rural health facilities in coastal Tanzania randomized to receive or not receive anti-malarials only 24 (2.4%) developed malaria; investigators identified only 3 cases of bacteremia and 1 case of urinary infection, along with 238 viral pathogens in stool and/or nasopharyngeal swabs [6]. There were no severe adverse events related to untreated illness, and no deaths. Among 1000 children presenting with fever at outpatient clinics in another study in both urban and rural mainland Tanzania, 71% had viral infections, and in most children the fever was self-limiting [7]. A randomized controlled cross-over study in Tanzania supported the safety of withholding artemisinin-based combination therapy (ACT) to mRDT negative patients receiving care in the community [8] and a study in Zambia found that only 8.2% of mRDT-negative children treated in the community with an antipyretic alone continued to have fever or were reported by the caregiver to be unwell at the time of the follow-up visit (day 5–7) [9]. These results suggest that in cases of febrile illness among children without danger signs, and with no other obvious cause of fever, expectant management at home can be safely used without a follow-up visit to the CHW on Day 3.

Therefore, current guidelines instructing CHWs to universally advise caregivers of children with uncomplicated fever to return on Day 3, even in the absence of continued fever or other signs, may be unwarranted and may create a substantial additional burden on CHW and caregiver time and resources. The extensive iCCM package that is delivered by CHWs, who are often volunteers, leads to a substantial workload and opportunity costs (i.e. limited time available to spend on income-generating activities). Caregivers often have to travel long distances by foot to reach a CHW, preventing them from engaging in income-generating activities as well.

One possible change to the current guidelines would have the child’s caretaker advised to return with the child for reassessment only in instances where signs persist or worsen. In order to provide the evidence base for such an update to global iCCM guidelines, we have designed a cluster-randomized, community-based trial to examine the non-inferiority of reassessment of RDT-negative febrile children without danger signs only in cases where signs do not resolve, compared with universal follow-up (independent of sign resolution), as recommended by WHO.

## Methods

### Aims

The primary aim of this study is to determine if the likelihood of clinical deterioration (“failure”) among children between 2–59 months of age in Tanganyika Province, DRC that present with an uncomplicated fever to CHWs is similar between those advised universally to be followed up on Day 3 and those advised for follow up only if illness does not resolve. At seven days post-enrollment, children are considered as “failed” if they present with persistent fever, persistent illness, or decline of health status (e.g. hospitalization, presenting danger signs, or death). We hypothesize that the proportion of children that meet this primary outcome determination under current guidelines (“universal”) is 5% and that the true rate of failure in the condition under study (“conditional”) is 6%. The null hypothesis is that the conditional follow-up approach is inferior and that children in this group will yield a failure rate at least 4% higher than that of the universal follow-up group. Conditional follow-up will be considered non-inferior to universal follow up if the upper bound of a one-sided 95% confidence interval around the absolute difference in outcome rate (conditional follow up minus universal follow up) does not exceed 4%.

Secondary aims of the study include describing the clinical presentation, care-seeking patterns, and outcomes among children that are classified as “failed” at Day 7, estimating the frequency of scheduled and spontaneous visits among all children, hospitalization and death rates among all children, and longer-term outcomes (based on data collected during Day 14 and Day 28 house visits) of children that were classified as “failed” at Day 7. In addition, a qualitative portion of the study with a limited sample size will look at CHWs’ and caretakers’ perceptions of the conditional vs. universal follow-up message.

Enrollment for this study was initiated in October 2015 and will be completed by the end of 2016. The study is a collaboration between the International Rescue Committee (IRC) and the Johns Hopkins Bloomberg School of Public Health.

### Study site and population

This study is implemented in Tanganyika Province in the southeastern DRC. With a density of 18 inhabitants per square kilometer, Tanganyika has endemic malaria transmission and low access to health care services due to geographic barriers and a lack of financial resources of those seeking care. To address these barriers, the International Rescue Committee (IRC), with Ministry of Public Health oversight, is currently implementing the Canadian International Development Agency-funded and WHO-administered Rapid Access Expansion (RAcE) program, a 4-year effort to train and deploy CHWs to deliver iCCM across six countries, including in 11 health zones in Tanganyika Province. Burden of fever in this setting is high: 2014 RaCE program data indicate that CHWs in seven of the health zones evaluated a total of 79,000 cases of fever in children under five, 13,000 of whom were mRDT negative.

The non-inferiority trial is being conducted in two RaCE-participating health zones with a total under-five population of ~168,000 (Kalemie and Nyemba, under-five populations of 94,000 and 74,000, respectively). Within each zone, 14 health areas are included, each having both a health center, and a team of associated CHWs (total 258; approximately 5–10 per area; average 104 (±81) children per CHW) who work within that particular health area. The CHWs are men and women with at least primary school education level, who are literate and locally resident, elected by the community, and have received five days of training on the iCCM algorithm as per DRC Ministry of Public Health guidelines. Medications (e.g. ACT, amoxicillin, oral rehydration solution (ORS), zinc, paracetamol, and ACT suppository for severe malaria cases requiring referral) and supplies are distributed to CHWs on a monthly basis, and the care they provide to children in their community is free of charge.

### Randomization

The unit of randomization in this trial is the health area (*n* = 28), and CHWs working within a particular health area are thus allocated to the same group (either “universal” or “conditional” follow-up advice given to caretakers of eligible children); all children enrolled by CHWs within a health area constitute the study cluster. Allocation to the “universal” versus “conditional” arm was done via restricted randomization whereby units were balanced on zone and health area estimates of 1) population size, 2) prior 6-month likelihood of mRDT-negative febrile children (number of children mRDT negative/under-five population), and 3) geographic distance from CHW to zonal health center. Of all possible randomization sequences (*n* = ~40.1 million, generated using STATA version 13.1), 43,504 sequences were balanced on zone ***and*** had ratios of the above three indicators between 1/1.05 and 1.05. After confirming the validity of the restriction procedure (approximately equal probability of any two units being assigned to the same or different arms), one of these sequences in the restricted subset was selected at random. It was not possible to mask the clustered allocation from participants, CHWs, or data collectors).

### Sample size

To calculate sample size, we set the outcome rate in the “universal” follow-up group to 5%, and assumed that the (true) corresponding outcome rate in the “conditional” follow up group is no more than 6%. For the purposes of concluding that the “conditional” follow up is non-inferior to the “universal” follow up approach, using the simple approach outlined by Blackwelder [10] and extending the methods of Hayes and Bennett [11], we first estimated the coefficient of variation (k) of the true failure rate across our available health areas; we conservatively estimated that k is 0.35, implying that the true cluster-specific failure rates vary between 1.5 and 8.5% (i.e. 5%*(1 ± 2 k)). Next, utilizing data from the first six months of 2015, aggregated from monthly reports from CHWs working in Kalemie and Nyemba health zones, we anticipated that approximately 493 negative mRDT cases would occur per month over 28 health areas. We estimated that about 70% of these mRDT negative cases would be eligible (i.e. excluding those cases with danger signs or other CHW treatable conditions [pneumonia, diarrhea]). Given the above assumptions, and desiring 12 complete months of enrollment, the estimated average cluster size over that anticipated timeframe is equal to (70%*493*12)/28 = 148. Assuming that about 10% of the eligible children will not participate or will be lost to follow up (reducing this yield to 148*.09 = 133, the total number of health areas required to detect (with 80% power) non-inferiority of the “conditional” follow up approach given the above parameters was 24, (12 per group). Rather than exclude just 4 of our available 28 health areas, we decided to include all 28, increasing the number of clusters to 14 per group (this increased our estimated power to 86.8%), and leading to an initial estimated final enrollment of 3730 children. After approximately 6 months of actual study enrollment, however, our actual observed per-cluster yield had increased to 152, leading to a revised estimate of the total enrollment; by the completion of the enrollment period, we will enroll approximately 4270, or 2135 children per group.

### Eligibility and intervention

All CHWs were trained on the study protocol including the appropriate follow up message for children with uncomplicated fever depending on study arm allocation, and the eligibility criteria for study inclusion. CHWs identify eligible children while conducting routine iCCM services in their communities. Assessment of children presenting for care under iCCM guidelines includes examining the child, asking the caregiver about signs of illness (e.g. danger signs, diarrhea, fever, cough, screening the child for malnutrition by measuring the mid-upper arm circumference [MUAC] of the child), and (rapid) testing for malaria. Some reported signs (such as cough or difficulty breathing) will prompt the CHW to look for chest in-drawing and measure respiratory rate. Children ***ineligible*** for this study are those the CHW determines to have one or more danger signs and/or malaria, pneumonia, or diarrhea; those with danger signs are immediately referred to the nearest health centers, while the others are treated and advised to return on Day 3. The remaining febrile children (i.e. those with neither danger signs nor a CHW-treatable illness) are eligible for enrollment. Such eligible children are provided an antipyretic and caretakers are advised if signs should worsen in the coming days, they should immediately seek care at the health center. Finally, the CHW provides study-arm specific advice about when to return for a follow up visit:

1. In the “universal” study arm, CHWs advise all caregivers to come back with the child on Day 3.
2. In the “conditional” (intervention) study arm, CHWs advise caregivers to come back with the child on Day 3 only if signs remain the same, or worsen. Caregivers are told that if they determine that the child is in good health, they do not need to return.

### Consent and enrollment

The CHW notifies his/her health area data collector about the identification of the eligible child. On Day 7 after identification, the data collector and the CHW jointly visit the child’s household to formally conduct an oral informed consent process, enroll the child, conduct the day 7 assessment, and record outcome and covariate data.

Upon first arriving at the home, the data collector reads aloud a consent script explaining the study, the CHW assessment procedures, and data collection activities, and answers any questions the caregiver might ask. If the caregiver verbally agrees to study participation, the data collector records vital status, axillary temperature, respiratory rate, and MUAC, and the CHW follows the iCCM algorithm to assess the child and query the caregiver about the child’s signs. Specifically, the CHW asks the mother if the child has cough, fever, diarrhea or any problem, and assesses the child for danger signs (unable to drink or breastfeed, vomiting everything, convulsions, loss of consciousness, chest-in drawing), warning signs (low MUAC, persistent illness, difficulty breathing/wheezing, signs of possible anemia), signs of malnutrition (visible signs of wasting, edema of lower limbs), and other signs (diarrhea, with or without blood in stool, generalized rash, and mother’s report of fever). In the event of reported fever by the caregiver, the CHW will check for malaria using a mRDT (and provide treatment, as necessary); if cough is reported by the caregiver the CHW will count the child’s respiratory rate to assess pneumonia. The data collector records all information from the assessment and also administers a structured questionnaire to elicit information on infant, maternal/paternal, and household variables, as well as on care-seeking behaviors.

Finally, information about the child’s initial visit to the CHW and any interim visits to the CHW (i.e. between identification of eligibility and the Day 7 assessment visit) is extracted by the data collector from the CHWs iCCM records. This extraction process provides an additional opportunity for the data collector to confirm that the initial eligibility assessment by the CHW was correctly done; permission to extract this information from the iCCM records is included in the consent process described above.

### Follow up of sick children

If the caregiver reports that the child has a fever, or if the CHW determines that the child has any danger signs, or has to be treated for malaria, diarrhea, or pneumonia during the follow up visit on Day 7 (i.e. child meets the primary outcome definition), an additional follow up visit is planned on Day 14 for the child. At this second home visit, the CHW and data collector again conduct the same health assessment as described above and the outcome of the visit is recorded. As before, if a child at a Day 14 visit is determined to have fever, any danger sign, or one of the three CHW-treatable illnesses, the appropriate treatment and/or or referral advice is provided, and a third visit is scheduled for Day 28. Thus, sick children are followed up at Day 14, and Day 28, if necessary. The vital status (alive/died) of all enrolled children is recorded by the data collector 30 days after initial enrollment, regardless of number of follow up visits conducted.

### Quality control and supervision

In order to ensure that all eligible children are correctly identified, data collectors check the registers of CHWs in the health area where they work on a weekly basis.

Quarterly meetings are organized for all data collectors and research support staff to provide feedback based on the data collected, to address challenges experienced by the data collectors in the field and to identify best practices. In addition, each data collector receives a quarterly individual supervision visit in the health area where (s)he works. During this visit, the Research Manager accompanies the data collector on a Day 7 visit to observe and evaluate the data collector while at work and to provide coaching and feedback in order to strengthen the data collector’s capacities. In addition, the Research Manager conducts spot checks of CHW registers working in the data collector’s health area during the individual supervision visit to ensure that all eligible children are identified by the CHWs and data collectors.

### Data management

All data are collected on paper forms by data collectors. Data collectors use an extraction form to transfer information from the CHWs iCCM records (i.e. register, individual sick child form) for the enrolled child. Other forms are filled out during household visits with enrolled children on Day 7, and, if necessary, on Day 14 and Day 28. Each enrolled child receives a unique six-digit code (“case id”) in order to ensure a linked chain of forms, as well as to protect the child’s and caregiver’s identity. Forms are checked for accuracy and completeness by the Research Manager and a Data Officer prior to data entry in Kalemie. All data forms are double-entered into a secure online database (REDCap) [12] using customized data entry screens, with built-in validation checks. De-identified analytic files are constructed from the raw REDCap database files.

### Analysis

**Fig. 1:**
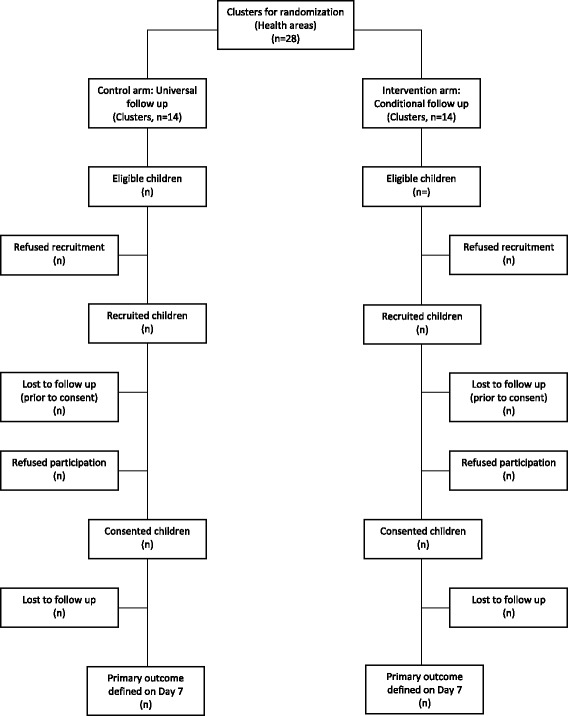
Trial design flowchart

All children enrolled and providing available data from the follow-up visit on Day 7 will be included in the analysis. First, we will present descriptive information on the recruitment, enrollment, and follow up of children with uncomplicated fever using a participant flowchart (Fig. 1). We will then assess characteristics of enrolled children to determine the extent to which our randomization procedure at the health area level achieved balance across the study arms; these characteristics will include infant, maternal/paternal, household, and socio-economic variables. Variables that are imbalanced will be noted for possible inclusion in a subsequent adjusted analysis.

The next stage of the analysis will focus on the primary outcome, failure at Day 7 visit, which is defined as fever, diarrhea, pneumonia or decline of health status (e.g. hospitalization, presenting danger signs, or death). In each study arm, the failure proportion will be estimated as the number of children that “failed”, and the non-inferiority of the two approaches to follow up advice (“universal” vs “conditional”) will be assessed by comparing these proportions across the two groups. The difference in these rates (proportion of children that failed in the conditional follow-up group minus proportion of children that failed in the universal follow-up group) along with a one-sided 95% confidence interval will be estimated. If the upper bound of this confidence interval is less than 0.04, we will reject the null hypothesis and conclude that the conditional follow-up approach is not inferior to the universal follow-up approach. If we have noted variables that are imbalanced across the allocation groups, we will conduct adjusted analyses of the difference in proportion using multivariate binomial regression models with an identity link function. A priori specified sub-analyses will include those where the women and CHWs both report providing the per-protocol advice, those where fever is detected based on axillary temperature (rather than caregiver’s report), and stratified by number of days of fever reported (>2 vs < =2). Secondary outcomes described previously will be examined using descriptive analytic approaches.

### Monitoring

An 3-person Data Monitoring Committee was formed to periodically receive from investigators updates on enrollment progress and numbers of deaths and hospitalizations. As the invention consisted of a change in follow-up schedule only, and all participants received usual care (i.e. followed iCCM guidelines) from CHWs at follow up visits, there was no formal interim analyses conducted for safety, efficacy, or futility.

## Discussion

This study, expected to be completed by the end of 2016, is a cluster-randomized, community-based non-inferiority trial evaluating conditional follow up versus a universal follow up of children who present uncomplicated fever and seek care at a CHW site. The results from this study will provide an evaluation of the clinical deterioration (“failure”) of children between 2 and 59 months of age that presented with an uncomplicated fever to a CHW and subsequently received universal versus conditional follow up. Furthermore, the results from this study will provide information regarding the clinical presentation, care-seeking patterns, and outcomes among children that are classified as “failed” at Day 7, the frequency of scheduled and spontaneous visits among all children, hospitalization and death rates among all children, and longer-term outcomes (based on data collected during Day 14 and Day 28 household visits) of children that were classified as “failed” at Day 7.

If the conditional follow up of children with uncomplicated fever is shown to be non-inferior to the current WHO iCCM protocol prescribing universal follow up of children with uncomplicated fever by CHWs, this will suggest that current guidelines can be simplified, having significant implications for community-based case management of uncomplicated fever in the DRC, and in many other settings. Simplification of the current iCCM algorithm by recommending a conditional follow up for children with uncomplicated fever will reduce unnecessary follow-up visits and reduce the burden on both CHWs and caregivers. Results will be interpreted in conjunction with a similarly designed trial testing the same hypothesis being conducted concurrently in Ethiopia.

## Abbreviations

ACT: Artemisinin-based combination therapy
CHW: Community health worker
DRC: Democratic Republic of the Congo
iCCM: Integrated community case management
IRC: International Rescue Committee
mRDT: Malaria Rapid Diagnostic Test
MUAC: Mid upper arm circumference
ORS: Oral rehydration solution
RAcE: Rapid Access Expansion program
WHO: World Health Organization
